# Comparing Urethral Stricture Rates Following Bipolar and Monopolar Transurethral Resection of the Prostate: A Retrospective Study

**DOI:** 10.7759/cureus.73548

**Published:** 2024-11-12

**Authors:** Anna Akpala, Ahmed Warda, Serene Batson-Patel, Saloni Bhattacharyya, Adebiyi Damola, Ahmed Farag, Amar Manandhar

**Affiliations:** 1 Urology, Queen Elizabeth Hospital Birmingham, Birmingham, GBR; 2 Urology, Northampton General Hospital, Northampton, GBR; 3 Urology, Bedford Hospital NHS Trust, Bedford, GBR; 4 Urology, George Eliot Hospital NHS Trust, Nuneaton, GBR; 5 Urology, Kettering General Hospital, Kettering, GBR; 6 Urology, University Hospitals of Birmingham, Birmingham, GBR

**Keywords:** benign prostatic hyperplasia (bph), bipolar transurethral resection, monopolar transurethral resection of prostate, transurethral resection of prostate, urethral stricture (us)

## Abstract

Aim

The aim is to compare the incidence of urethral strictures and other complications following monopolar and bipolar transurethral resection of the prostate (TURP).

Method

We conducted a retrospective study to compare patients who underwent bipolar TURP with those who underwent monopolar TURP between 2017 and 2023. The collected data included demographics, age, history of urethral stricture, prostate size, operation duration, and postoperative complications, such as blood transfusion, transurethral resection (TUR) syndrome, and other relevant data points.

Results

The COVID-19 pandemic significantly affected the number of surgeries performed. A total of 572 patients who underwent TURP at our center during this period were identified, 302 of whom underwent monopolar TURP, and 270 underwent bipolar TURP.

Bladder neck stenosis was more frequently identified in the monopolar group compared to the bipolar group (1.99% (6) vs. 0.7% (2)). In the monopolar group, 6.62% (20) of the patients had strictures compared to 4.07% (11) in the bipolar group; however, this difference is not statistically significant. The bipolar group had a higher incidence of urinary incontinence (5.6% (15) vs. 3.3% (10)), whereas the monopolar group had higher readmission rates (18.8% (57) vs. 13.7% (37)) and a higher frequency of delayed trial without catheter (TWOC) (84% (254) vs. 75.9% (205)).

Conclusion

We believe that our findings contribute towards resolving the debate between stricture complication rates in monopolar versus bipolar TURP. Our analysis revealed no statistically significant differences in stricture rates between the two groups. However, we noted differences in other complications, such as higher rates of urinary incontinence in the bipolar group, whereas the monopolar group had increased rates of readmission and bladder neck stenosis.

## Introduction

Benign prostatic hyperplasia (BPH) is a histological condition characterized by the proliferation of the connective, stromal, and glandular tissues of the prostate [1]. The incidence and prevalence of BPH, along with subsequent lower urinary tract symptoms resulting from the compression of the urethra by the prostate, has increased widely, owing to the aging world population [1]. BPH can lead to bothersome lower urinary tract symptoms, such as frequency, urgency, nocturia, weak stream, intermittency, straining, and incomplete bladder emptying, and may eventually lead to urinary retention. This significantly impacts the quality of life of patients.

Modalities of treatment include watchful waiting with lifestyle modifications and pharmacological and surgical management. The modality of treatment chosen will be in a stepwise approach and gauged using the International Prostate Symptom Score (IPPS) or American Urological Association (AUA) symptom questionnaire [2], patient preference, and fitness for intervention.

BPH refractory to conservative and pharmacological management are treated surgically.

Currently, the surgical management of BPH ranges from minimally invasive techniques such as prostatic urethral lift (Urolift), prostatic artery embolization, paclitaxel-coated prostatic balloon dilation, Aquablation, water vapor or steam infusion therapy (REZUM), and a temporarily inserted nitinol device (iTind) to more invasive techniques such as transurethral resection of the prostate (TURP), holmium laser enucleation of the prostate (HoLep), green light laser enucleation of the prostate (GreenLEP), and open prostatectomy [2].

Transurethral resection of the prostate (TURP), developed in the 1940s, is the most common BPH surgery and is still considered the gold standard for the surgical management of BPH [3].

The electrical current used in TURP can be either in the form of a monopolar or bipolar diathermy technique. Although it is considered a common urological procedure, it is not without risks. Transurethral resection (TUR) syndrome was a major concern in monopolar TURP until the evolution of bipolar TURP [4]. Bipolar resection may be slower [2] because it simultaneously coagulates as it resects but appears safer in terms of bleeding and TUR syndrome. Resection is performed in saline compared to monopolar, which is performed in glycine, a hypotonic fluid that risks the development of TUR syndrome. Studies have revealed conflicting results in stricture complication rates between monopolar and bipolar TURP. The EAU guidelines 2024 do not appear to differentiate the complication rates between monopolar and bipolar TURP. However, some studies have suggested that there are more postoperative strictures in bipolar TURP than in monopolar TURP [5,6,7,8], so we set out to investigate this as our center has largely migrated to the bipolar TURP system.

## Materials and methods

This retrospective study was performed at the Queen Elizabeth Hospital Birmingham (QEHB), United Kingdom, and included all patients who underwent monopolar and bipolar TURP between January 2017 and January 2023. A total of 572 patients were retrospectively assessed within the timeframe.

The inclusion criteria included all patients who underwent either monopolar or bipolar TURP at the QEHB between 2017 and 2023. A total of five patients who underwent both monopolar and bipolar TURP were excluded.

The data collected included demographics, age, previous history of urethral stricture, prostate size, duration of operation, level of expertise of the operator, that is, registrar or consultant, post-operative complications such as blood transfusion, TUR syndrome, readmission, duration of catheterization before trial without catheter (TWOC), readmission, urethral stricture based on anatomical location, that is, penile urethral stricture, bulbar urethral stricture, prostatic urethral stricture, and bladder neck stenosis. The data was obtained from the patient's electronic records following approval from the clinical audit registration and management system of the department; these were then entered into an Excel sheet, and the data was analyzed after a quality assurance review.

Statistical analysis of the data was carried out with R version 4.2.1 (R Foundation for Statistical Computing, Vienna, Austria, https://www.R-project.org/), and Pearson correlation was used to check the correlation between the variables. Statistical significance was set at a value of p<0.05.

## Results

After reviewing all the patient records between January 2017 and January 2023 and applying the exclusion criteria stated above, we had 302 patients who had monopolar TURP and 270 patients with bipolar TURP. 

Demographic data

**Figure 1:**
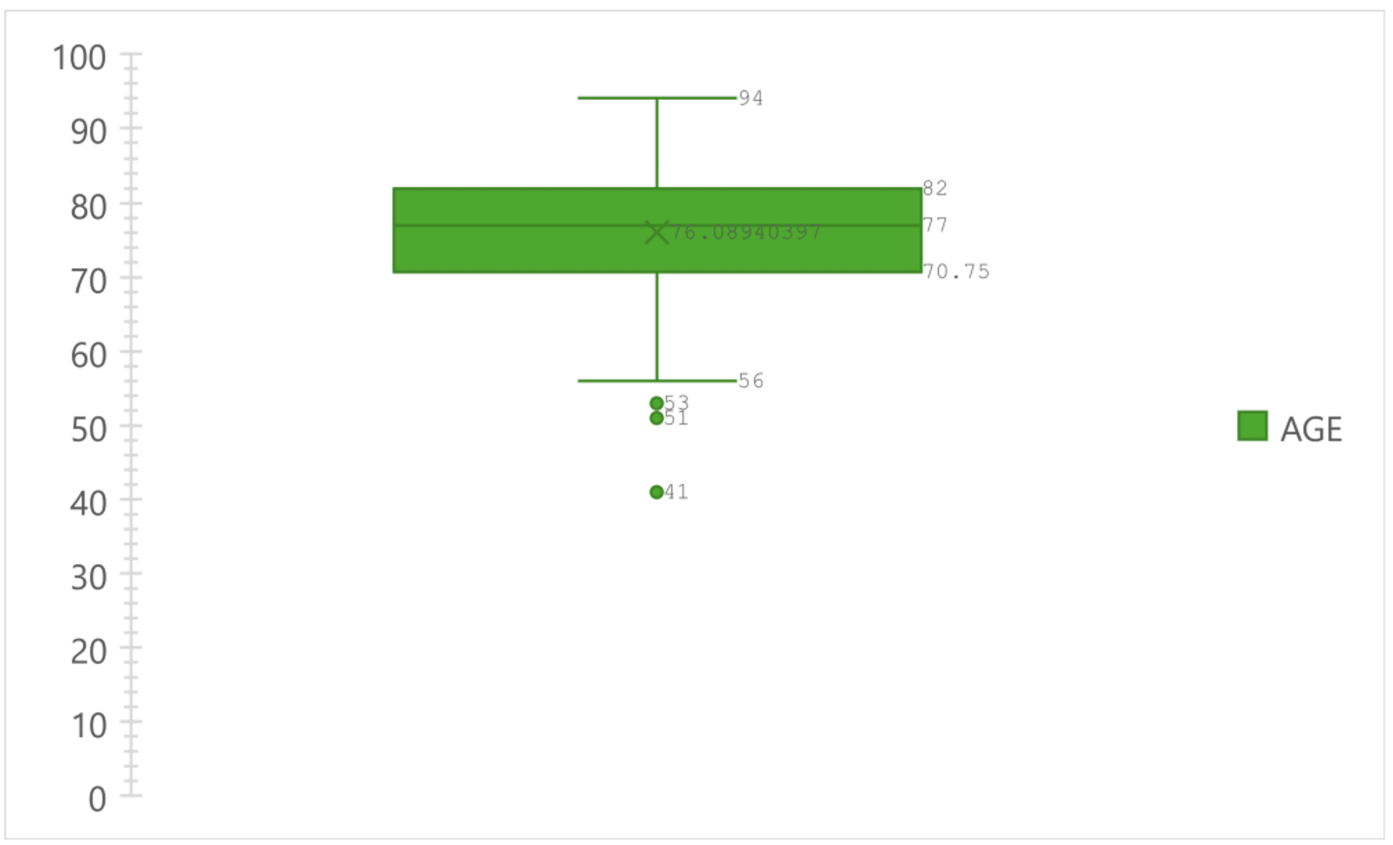
Boxplot showing age distribution and mean age of the patients in the monopolar group expressed in number (N)

**Figure 2:**
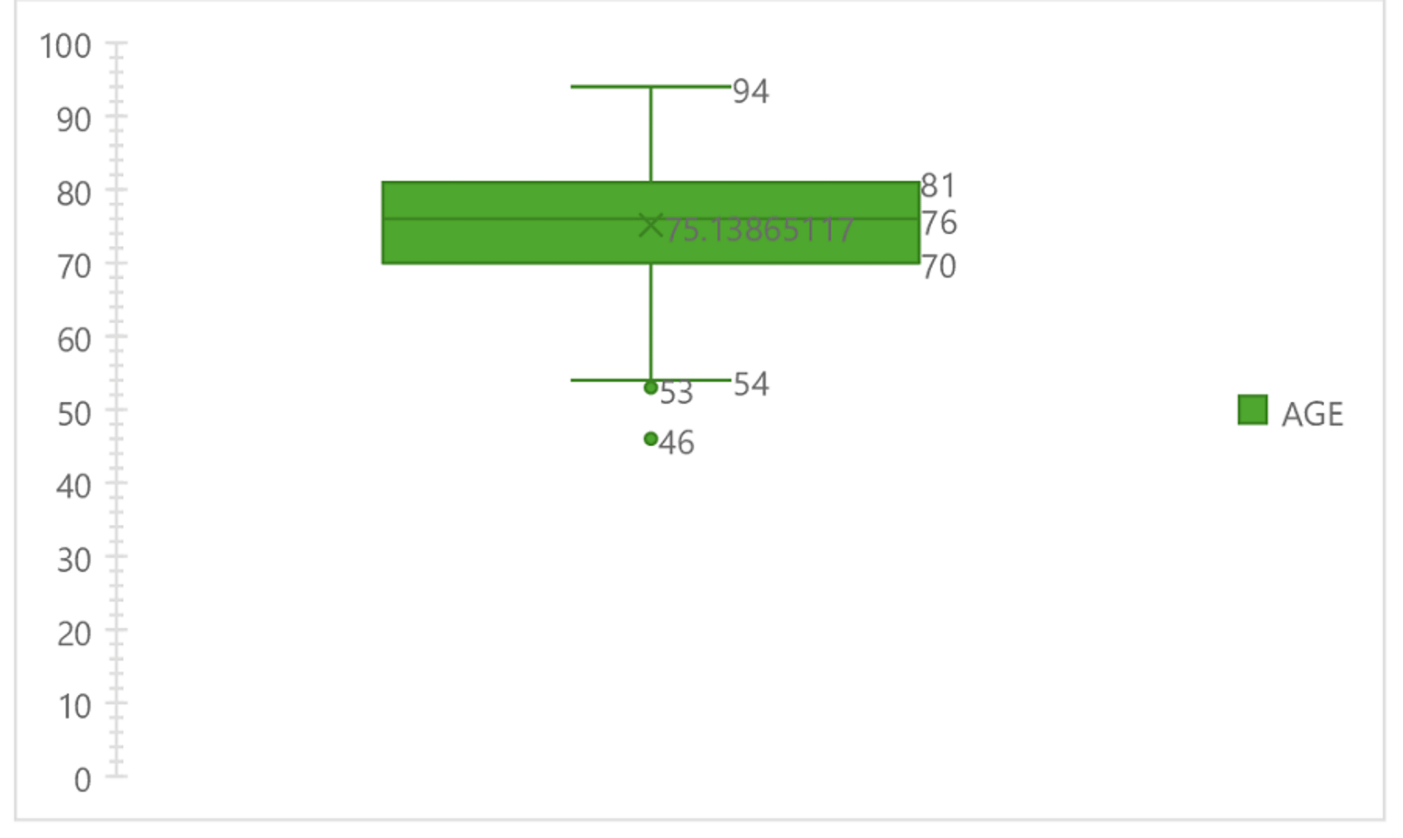
Boxplot showing age distribution and mean age in the bipolar group expressed in number (N)

The baseline data were similar between the two groups, which comprised 572 evaluable patients. The mean age is 76.1 for the monopolar group and 75.1 for the bipolar group, as shown in Figures 1, 2.

Perioperative measurements

Perioperative measurements as listed in Table 1.

**Table 1:** Perioperative measurements Pearsons's product-moment correlation analysis was performed to check the correlation between the individual variables with stricture rates significant at p<0.05 expressed in numbers (N) g: grams; min: minutes; cor: correlation

Variable	Monopolar (n=302)	Bipolar (n=270)	P-value	Cor
Prostate size (g)			0.1176	0.3145
<50	156	122		
51-100	133	110		
101-150	11	31		
>150	2	7		
Duration of operation (min)			0.4112	0.1689
<60	274	190		
>60	28	80		
Bladder neck incision	61	30	1	0
Expertise of operator			0.4346	0.1601
Consultant	142	129		
Registrar	160	141		
Previous stricture	21	20	1	0

Prostate size

Preoperative prostate volume measured on ultrasound and computed tomography (CT) scan showed 51.66% (156) of prostates in the monopolar group weighed <50g in comparison with 44.04% (133) of the same size in the bipolar group. Conversely, only 0.66% (2) of the prostate size in the monopolar group was >150 g, compared to 2.59% (7) in the bipolar group. We sought to correlate prostate size and stricture rates and found no correlation (p = 0.1176) using Pearson’s product-moment correlation.

Previous history of urethral strictures

We assessed for a history of urethral stricture prior to TURP and sorted to correlate this with further strictures following TURP. Only 6.95% (21) of patients in the monopolar group had a previous history of urethral stricture compared with 7.40% (20) in the bipolar arm. Using Pearson's product-moment correlation, there was no statistically significant correlation between previous urethral strictures and new strictures post-TURP (p = 1). 

Duration of operation

In the monopolar group, 90.73% (274) had their operations lasting for less than one hour, while the remaining patients in this group lasted for an hour or more. In the bipolar group, the results were not similar, with 70.55% (190) under one hour and 29.45% (80) more than one hour. Approximately one-third of the patients in the bipolar group underwent resections for more than one hour; however, the overall incidence of urethral stricture was lower in this group (11.1% (11) vs. 20.2% (20)) compared to the monopolar group. Using Pearson's correlation, there was no statistically significant correlation between the duration of resection and the stricture rate (p = 0.4112).

Bladder neck incisions

In the monopolar group, 20.2% (61) of the patients had concurrent bladder neck incision (BNI) at the time of surgery, while only 11.1% (30) of the patients in the bipolar TURP group had the same. The analysis showed no statistically significant correlation between bladder neck incision and stricture rate (p = 1).

Expertise of operator

The procedures were performed by either consultants or their registrars. In the monopolar group, 47.02% (142) of the procedure was performed by a consultant, which was similar to the 47.78% in the bipolar group. Statistical analysis showed no significant correlation between operator and stricture rates (p = 0.4346).

Days to successful trial without catheter (TWOC)

The number of days required for catheter removal varied. Around 15.9% (48) of the patients in the monopolar group had a successful trial without catheter (TWOC) on day one postoperatively, compared to 24.1% (65) in the bipolar group. The median time for successful TWOC was the same in both groups, two to five days.

Complications

A variety of postoperative complications were identified in our population grouping. A list of these complications is presented in Table 2.

**Table 2:** Complications following TURP in our population grouping Pearson's product-moment correlation was performed to check the correlation between the type of TURP and readmission rate significant at p<0.05. Data expressed in number (N) and % cor: correlation; TURP: transurethral resection of the prostate

Variable	Monopolar (n=302)	Bipolar (n=270)	P-value	Cor
Readmissions	57 (18.9%)	37 (13.7%)	0.0663	-0.0766
TUR syndrome	1 (0.331%)	1 (0.370%)		
Urinary incontinence	10 (3.31%)	15 (5.56%)		
Post-op transfusion	2 (0.662%)	1 (0.370%)		

Readmissions

In our study, 60% (57) of all readmissions were from the monopolar group, while 40% (37) were from the bipolar group, but this had no significant statistical correlation (p = 0.0662).

Urinary incontinence

We observed that 3.31% (10) of the patients in the monopolar group developed urinary incontinence postoperatively, while 5.56% (15) of patients in the bipolar group became incontinent. This could potentially be explained by the fact that the bipolar group of patients had higher prostate volumes and thus a longer duration of operation beyond one hour, thereby increasing the risk of sphincteric injury and subsequent urinary incontinence.

TUR syndrome

In our study, there were no differences in TUR syndrome, as only one patient from each group developed it postoperatively.

Post-operation blood transfusion

There were no significant differences in postoperative transfusion rates between the two groups. Postoperative bleeding following TURP requiring blood transfusion occurred in only two patients (0.66%) in the monopolar group and one patient (0.37%) in the bipolar group.

Urethral strictures

Urethral strictures following TURP were further classified based on their anatomical location, as described above. A total of 20 patients in the monopolar group developed strictures postoperatively compared with 11 patients in the bipolar group, of whom six patients in the monopolar group developed bladder neck stenosis compared with only two patients in the bipolar group. Despite an almost 50% disparity between the two groups, statistically, this was insignificant (p = 0.4112). It can be inferred that bulbar urethral strictures were the most common type of stricture that developed postoperatively in both groups, as shown in Table 3.

**Table 3:** Urethral strictures complication Urethral strictures following TURP, data expressed in numbers (N), and %. Pearson's product-moment correlation is significant at p<0.05 cor: correlation; TURP: transurethral resection of the prostate

Strictures	Monopolar (n=20)	Bipolar (n=11)	P-value	Cor
Penile	3 (0.993%)	0		
Bulbar	9 (2.98%)	8 (2.96%)		
Prostatic	2 (0.662%)	1 (0.370%)		
Bladder neck stenosis	6 (1.99%)	2 (0.741%)	0.4112	0.1683

## Discussion

Urethral stricture rates

There are conflicting data in the literature regarding urethral stricture rates after bipolar and monopolar TURP. Tang et al. [9], in their systematic review, reported no significant differences in stricture rates between monopolar and bipolar TURP. This is supported by other studies [10-14] where no significant differences in stricture rates were found, suggesting comparable and acceptable rates. This is supported by the results of our study, in which no significant difference in stricture rates was found between monopolar and bipolar TURP (p = 0.4112). However, Pirola et al. [15] reported a higher rate of urethral strictures in monopolar TURP. In their systematic review, their focus was comparing TURP to enucleation and ablation, with both monopolar and bipolar TURP having higher rates of urethral stricture in comparison to enucleation and ablation techniques for prostate resection (3.8%, 2.1%, 1.7%, and 2.1%, respectively). Although no significant result was the conclusion by the authors between monopolar and bipolar stricture rates, the authors hypothesized that thermal injury to the urethra through currents utilized by monopolar and bipolar TURP can result in stricture rates, which could be mitigated through lubrication of the outer sheath [15]. In contrast, Elsaqa et al. [16] and Komura et al. [17] reported a higher stricture rate in bipolar TURP than in monopolar TURP. While Elsaqa et al. had a small sample to compare, their multivariate analysis reported that a larger prostate volume and longer operative time were associated with a risk of strictures and bladder neck contracture [16]. Similarly, Komura et al. reported a significant result in their randomized control trial for bipolar TURP urethral stricture rates (6.6% in monopolar TURP vs. 19.0% in bipolar TURP; P = 0.022) [17]. Interestingly, Komura et al. also found higher prostate volumes (>70 ml) and stricture rates in the bipolar group than in the monopolar group (20% in bipolar TURP vs. 2.2% in monopolar TURP; p = 0.012) [17], which supports the findings of Elasqa et al. [16]. However, our results showed no correlation between prostate size and stricture rates (p = 0.1176), although our grouping used a cut-off of 150 g for comparison, which could explain these differences. Overall, while some data suggest that a higher stricture rate in bipolar TURP could be due to a larger prostate volume, the majority of studies as well as our results support that there is no difference in postoperative urethral stricture rates between bipolar and monopolar TURP.

History of urethral strictures

However, it is important to note that a medical history of urethral strictures may increase the chance of those presenting post-surgery. Our study found no statistical significance between previous urethral strictures and new strictures post-TURP (p = 1). Often, studies excluded patients who had previous evidence of urethral stricture or bladder neck stricture when assessing stricture rate complications post-TURP [18], making the literature sparse and therefore difficult to compare. Future studies should consider previous stricture rates as a factor for new stricture formation post-TURP to allow for comparison. 

Length of surgery

Our results showed no significant difference between the length of surgery and stricture rate (p = 0.4112). The surgery duration being less than 60 minutes was comparable between our monopolar TURP and bipolar TURP groups, at 90.73% (274) and 70.55% (190), respectively. This is similar to the results found by Pirola et al. [15] in their meta-analysis, where no significant differences were found in stricture rates in relation to the mean duration of surgical time. However, most of their surgeries did not exceed 60 minutes, similar to our study [19]. Another meta-analysis also reported no significant differences between monopolar TURP and bipolar TURP for procedure duration but did not report its effect on urethral stricture rates [19]. Our results also agree with those of other studies [11,12] in which the duration of the operation was not correlated with stricture rates. In contrast, a case-control study investigating stricture rates with the bipolar TURP system found that a slow resection rate was the only risk factor associated with urethral stricture occurrence [20]. This result is similar to that of a large retrospective study, where multivariate analysis showed that prolonged operative time was a significant risk factor for urethral stricture development (p<0.001) [21]. Other studies in the literature suggest that a prolonged operation time leads to stricture formation after TURP [22], especially surgeries lasting over 60 minutes [23], suggesting prolonged resectoscope activity resulting in inflammation and ischemia in the urethra [24]. Therefore, while our results showed no significant difference in the length of surgery and urethral stricture rate, this is highly likely because most surgeries were performed for less than 60 min, as the literature strongly suggests that a longer resection time is linked with urethral stricture rate formation due to inflammatory mechanisms.

Prostate size 

In our study, prostates that weighed less than 50 g were comparable between the monopolar TURP and bipolar TURP groups (51.60% (156) vs. 44.04% (133)). Few prostates weighed over 150 g, and this was larger in the bipolar TURP group than the monopolar TURP group (2.59% (7) vs. 0.66% (2)). We found no correlation between prostate size and the urethral stricture rate (p = 0.1176). However, studies have found that a larger prostate volume is associated with a higher rate of strictures, with a higher tendency for bipolar TURP [16,17]. Komura et al. found no significant differences in stricture rates with prostate volumes less than 70 ml; however, with prostate volumes >70 ml, there was a significantly higher stricture rate in the bipolar group than in the monopolar group (20% vs. 2.2% in monopolar TURP; P = 0.012) [17]. Elsaqa et al. theorized that this is likely due to the larger prostate requiring longer operative times, which can increase the risk of strictures as well as bladder neck contractures [16]. The largest prostate volume in our analysis was >150 g; seven of these patients were in the bipolar group and two were in the monopolar group. Despite the larger prostate volumes, these patients had no stricture complications post-TURP. The difference between our results and the literature could be due to a paucity of numbers in the larger prostate groups or the use of different prostate volume parameters to compare stricture rates, as the literature uses prostate volumes of <50 g or >50 g as a cut-off for small or large prostate, respectively. Therefore, despite our finding of no difference in prostate size and stricture rates, there may be a link due to associations with longer operative times.

Bladder neck incisions

We found no statistically significant correlation between bladder neck incisions and the development of strictures (p = 1). Unfortunately, no studies have demonstrated a link between these two factors, necessitating further research in this area.

Expertise of the operator

This study found that there was no significant correlation between operator expertise and stricture rates post-TURP (p = 0.4346). This may again be linked to the findings reported in the length of surgery section, as trainees are more likely to require more time to complete TURP [25].

TWOC

Our study found that there was a higher rate of delayed TWOCs in those who underwent monopolar TURPs (84%(254)) compared to those who underwent a bipolar resection (75.9%(205)). In this study, a delayed TWOC is defined as a TWOC beyond one-day post-op. These results are congruent with the systematic review by Bruce et al. [26], who found that bipolar TURP had a significantly shorter duration of catheterization compared to monopolar TURP (MD 11.92, 95% CI -0.10 to 23.94, p = 0.05); however, it was noted that there was high heterogeneity between the included studies (I2 = 96%, p<0.00001). As the level of heterogeneity was considerable, it should be questioned whether these studies were suitable for inclusion in a meta-analysis and if sub-group analysis would have been better [27]. This certainly appeared to be a concern in Tang’s systematic review [9], which noted that they would be unable to perform a meta-analysis on catheterization time in monopolar compared to bipolar TURPs because of their similar heterogeneity (I2 = 94%, p<0.00001). Unlike Bruce et al. [26], Sinha et al. [12] found no significant differences in the duration of catheterization for patients who underwent monopolar vs. bipolar TURP in their literature review. This finding was synonymous with a study by Fagerström et al. [28], who reported no significant differences in the length of catheterization in hours between the monopolar (mean = 20, median = 13-262) and bipolar (mean = 20, median = 13-115) groups.

The National Institute for Health and Care Excellence (NICE) [29] outlines that the claimed benefit of the bipolar system for TURP is an earlier catheter removal time, which can have massive implications on the length of hospital stay and, by extension, the cost of care. However, it must be noted that there was no significant statistical difference between the monopolar and bipolar groups in our study and that numerous other studies were unable to confidently ascertain if there was a significant difference in TWOC time [9,12,28].

Readmissions

Although a higher rate of admissions was from the monopolar TURP group compared to the bipolar TURP group (18.9% (57) vs. 13.7% (37)), there was no significant difference (p = 0.0662) between them. Patients who have undergone TURP may be readmitted to the hospital for a plethora of reasons, not limited to infection, hemorrhage, catheter problems, and urinary retention [28]. Our findings conflict with those of the randomized controlled trial (RCT) by Fagerström [28], who found that there were fewer readmissions in the bipolar group than in the monopolar (5 vs. 14, p<0.011). This RCT was further supported by NICE [29], who stated that evidence from studies has found that there were fewer readmissions in patients who underwent bipolar TURP using PLASMA than in those who underwent a monopolar TURP. Consequently, they recommended the use of a bipolar system over a monopolar system.

TUR syndrome

Although there is a consensus that the risk of TUR syndrome is higher in monopolar TURP when compared to bipolar TURP, a similar percentage of patients experienced this complication in this study (0.331% (1) vs. 0.370% (1)). For example, Tang et al. [9] and Bruce et al. [26] found that the risk of experiencing TUR syndrome was significantly higher in patients who underwent monopolar TURP than in those who underwent bipolar TURP. Tang et al. [9] concluded that there was a significant difference in the occurrence of TUR syndrome between the types of TURP (risk difference = 0.02; 95% CI, 0.01-0.03; P = 0.0004). However, they also noted that individual trials often reported no significant differences, much like ours. Despite this, Tang et al.’s [9] results were further supported by Bruce et al. [26] (OR 4.28, 95% CI 1.17 to 15.60, p = 0.03) and Omar et al. [30] (risk ratio (RR) 0.12, 95% confidence interval (CI) 0.05-0.31, p<0.001), which found the odds and risk of TUR syndrome to be higher in monopolar groups. A Cochrane review [17] in 2020 concluded that the use of bipolar TURP probably reduced the risk of TUR syndrome compared to monopolar TURP (relative risk (RR) 0.17, 95% CI 0.09-0.30; participants = 6,745, RCTs = 44; moderate certainty of evidence); hence, further randomized controlled trials investigating this outcome between the two methods were not warranted.

Multiple authors [9,25,30] have stated that bipolar TURP utilizes an irrigation fluid that is more physiologically similar to the human body compared to the hypotonic, non-conductive fluids used in monopolar TURP. Hence, dilutional hyponatremia does not occur in bipolar resection. However, it is important to note that TUR syndrome can still occur in patients who undergo bipolar resection, as evidenced by these studies and the present study. This is explained by Bruce et al. [26], who noted that the risk of TUR syndrome is also associated with the total operative time, amount of prostate resected, and blood loss, which can affect the amount of fluid shift and amount of irrigation utilized.

Urinary incontinence

Around 3.31% (10) of the patients who underwent monopolar TURP developed urinary incontinence post-operatively compared with 5.56% (15) who underwent bipolar TURP, although there was no significant difference between these groups. This finding was supported by Alexander et al. [25], who concluded that there is a similar risk of urinary incontinence at 12 months between monopolar and bipolar resections (RR 0.20, 95% CI 0.01-4.06; participants = 751; RCTs = 4), although there was low certainty of evidence surrounding this, thus demonstrating that more research should be carried out in this area in the future, perhaps a prospective randomized controlled trial for high-quality evidence.

Post-op transfusions

This study found no significant difference between monopolar TURPs and bipolar TURPs regarding postoperative transfusions (0.662% (2) vs. 0.370% (1)). These findings are supported by those in systematic reviews by Tang et al. [9] (risk difference 0.02; 95% CI, 0.01-0.04; P = 0.0005) and Bruce et al. [26] (OR 1.68, 95% CI 0.52 to 5.41, p = 0.39 I2 = 18%, p = 0.30). Interestingly, there was a significant difference noted in Fagerström’s RCT [28], which noted ten transfusions in the monopolar group compared to four in the bipolar group (p<0.01). Similarly, the Cochrane review [25] concluded that bipolar TURPs probably reduced the need for blood transfusions (RR 0.42, 95% CI 0.30-0.59; participants = 5727, RCTs = 38) with a moderate certainty of evidence and that further research in this area was not required. It is important to acknowledge that the Cochrane review [25] was published more recently than Bruce [26] and Tang’s [9] systematic reviews. Consequently, further research is likely to have been conducted since the latter’s systematic reviews, which are included in the Cochrane review [25], thus making it more accurate. National Institute for Health and Care Excellence (NICE) [29] has also stated that fewer patients required blood transfusions in the PLASMA bipolar group than in the monopolar TURP groups.

Limitations

Although there are strengths to this study, it is also important to acknowledge its limitations, the main one being its design. Because our study was retrospective, it is susceptible to numerous types of bias, such as selection bias. Additionally, our sample size included only 572 patients, although the introduction of bipolar TURPs has only occurred in recent years. A prospective study with power analysis would be well suited to determine an adequate sample size to detect significant differences. Additionally, we did not examine certain complications such as clot retention, sexual function, and quality of life scores, such as the International Prostate Symptom Score (IPSS), due to the lack of data in our clinical records. While we found no differences in prostate size and length of surgery in relation to the development of urethral stricture rates, the literature reports that larger prostate volumes and longer resections result in a higher urethral stricture rate [17,20]. With a larger sample size and a sub-group analysis of larger prostates, potentially significant results could have been obtained; however, this is difficult due to this lack within our population. Similarly, most surgeries took less than 60 minutes within our population, limiting our ability to explore this link further. Therefore, a multicentre study would increase the accuracy of the results obtained through larger sample sizes and varied populations, potentially allowing for links such as those mentioned previously to be uncovered, which may not have been found within this study itself. 

## Conclusions

With more advances in the surgical management of BPH, monopolar TURP appears to be fading away along with its adverse effects, such as TUR syndrome. From our study, more patients (20) from the monopolar group developed urethral strictures compared to the bipolar group (11); the monopolar group had a higher readmission rate and delayed TWOC in comparison to the bipolar group; however, this was of no statistical significance. The bipolar group had a higher incidence of urinary incontinence (5.6% (15) vs. 3.3% (10)), and this could be from more resections, therefore a higher risk of injury to the sphincter, as we found the highest prostate volumes in the bipolar group. Overall, we have demonstrated no significant statistical differences in adverse effects such as stricture rates, post-op transfusion, and readmission rates between the monopolar and bipolar TURP groups. Although HoLep is being considered the next generation “gold standard,” bipolar TURP remains the current gold standard in the surgical management of BPH due to its availability, cost, and low-profile complications.
